# Subnormal GM1 in PBMCs: Promise for Early Diagnosis of Parkinson’s Disease?

**DOI:** 10.3390/ijms222111522

**Published:** 2021-10-26

**Authors:** Samar K. Alselehdar, Monami Chakraborty, Suman Chowdhury, Roy N. Alcalay, Matthew Surface, Robert Ledeen

**Affiliations:** 1 Department of Pharmacology, Physiology, and Neuroscience, Rutgers, The State University of NJ, Newark, NJ 07103, USA; salselehdar@gmail.com (S.K.A.); mon.micro22@gmail.com (M.C.); sumanc080@gmail.com (S.C.); 2 Department of Neurology, Columbia University Irving Medical Center, New York, NY 10032, USA; rna2104@cumc.columbia.edu (R.N.A.); ms6015@cumc.columbia.edu (M.S.)

**Keywords:** early diagnosis, sporadic Parkinson’s disease, GBA variant of Parkinson’s, GM1 ganglioside, GD1a ganglioside, PBMCs, HPTLC, cholera toxin B

## Abstract

The fact that Parkinson’s disease (PD) pathologies are well advanced in most PD patients by the time of clinical elucidation attests to the importance of early diagnosis. Our attempt to achieve this has capitalized on our previous finding that GM1 ganglioside is expressed at subnormal levels in virtually all tissues of sporadic PD (sPD) patients including blood cells. GM1 is present in most vertebrate cells, is especially abundant in neurons where it was shown essential for their effective functioning and long term viability. We have utilized peripheral blood mononuclear cells (PBMCs) which, despite their low GM1, we found to be significantly lower in sPD patients compared to age-matched healthy controls. To quantify GM1 (and GD1a) we used high performance thin-layer chromatography combined with cholera toxin B linked to horseradish peroxidase, followed by densitometric quantification. GM1 was also deficient in PBMCs from PD patients with mutations in the glucocerebrosidase gene (PD-GBA), apparently even lower than in sPD. Reasons are given why we believe these results obtained with patients manifesting fully developed PD will apply as well to PD patients in preclinical stages—a topic for future study. We also suggest that these findings point to a potential disease altering therapy for PD once the early diagnosis is established.

## 1. Introduction

Parkinson’s disease (PD) has been termed the fastest growing neurological disorder in the world [1], progressing in synchrony with the increasing age span of individuals worldwide. The absence of treatment that would affect disease progression attests to the importance of early diagnosis. It has become evident that the low efficacy of treating PD patients is due at least in part to the late diagnosis and start of therapy. By the time of clear clinical diagnosis of PD half or more of the dopaminergic (DA) neurons of the nigrostriatal pathway have suffered viability decline or complete loss. Those DA neurons have received primary attention owing to their direct involvement in the movement disorders that constitute the clinical hallmarks of sporadic PD (sPD). However, the neuropathology of sPD is now recognized as much broader in scope, major cell loss also occurring in other brain regions and cell types. A prime example is the basal forebrain where the cholinergic neurons start to degenerate early in sPD and gradually worsen with the appearance of dementia [2]. The noradrenergic neurons in the locus coeruleus are among the first to suffer degenerative loss in sPD [3] while serotonergic neurons are also involved [4]. Moreover, neurons throughout the body are also affected before, during, and after the motor dysfunction sets in. Treatment of these diverse populations with their associated symptoms would clearly be more effective if commenced early enough to prevent the extensive neuronal losses destined to occur, especially if accompanied by disease-altering therapy that would terminate progressive pathological changes.

Numerous efforts have been made and are currently in progress to develop such early diagnosis based on a variety of behavioral and imaging methodologies as well as biochemical markers [5]. Some of these have proved to be reliable indicators of preclinical sPD symptoms but what is additionally needed is the connection of the indicator to appropriate therapy to effectively terminate disease progression at the early stage. We present here our findings on peripheral blood mononuclear cells (PBMCs) and their depressed level of GM1 ganglioside that shows promise as a reliable biochemical marker while also pointing to potential disease-altering therapy. GM1 along with its oligosialo-analogs occurs in numerous mammalian cell types and is especially abundant in neurons where it mediates a wide variety of regulatory and neuroprotective functions essential for effective signaling and long-term viability [6,7] (see Discussion). Our findings suggesting a systemic deficiency of GM1 in the brain and peripheral tissues of sPD patients point to this as a major contributor to PD pathogenesis and GM1 replacement as a promising therapy for disease alteration; some evidence that this is the case is already at hand (see Discussion) [8]. Here we show that PBMCs despite their low level of GM1 expression, show GM1 deficiency compared to healthy controls and also the subnormal levels characteristic of sPD tissues in general. The PBMCs employed in this study were obtained from PD patients well advanced in the disease but reasons are given why we consider it likely the same will be found in patients with pre-clinical PD (see Discussion).

In diagnosis and treatment of PD, it is beneficial to distinguish those patients with mutations in the glucocerebrosidase (GBA) gene from those with sporadic PD lacking such mutations; such mutations are found in 5–20% of PD cases and their symptoms and treatment options differ somewhat [9]. GBA encodes the lysosomal enzyme glucosylceramide hydrolase which showed lower activity in GBA homozygotes/compound heterozygotes and also GBA heterozygotes compared to non-carriers [10]. Mutations in this gene are the most significant genetic risk factors for sporadic PD. The clinical course of PD-GBA has been shown to be more aggressive with an earlier age of onset and a lower median survival time than sporadic PD [9]. The present study using a revised and simplified procedure shows GM1 levels in PBMCs from such PD-GBA patients to be subnormal like PD (including genetic cases) in general and even lower than in sPD.

## 2. Results

### 2.1. Clinical Characteristics of sPD and PD-GBA Patients and Healthy Control Subjects

Lipid analysis was completed for a total of 23 sPD patients, 13 PD-GBA patients, and 21 age-matched healthy controls, with males and females in each group. The demographical information and clinical assessment of all subjects are shown in Table 1 and Table 2. PBMCs from sPD and PD-GBA patients and controls were analyzed in two separate cohorts (as identified in Table 1 and Table 2).

### 2.2. Original Method: Analysis of GM1 and GD1a in PBMCs

**Figure 1:**
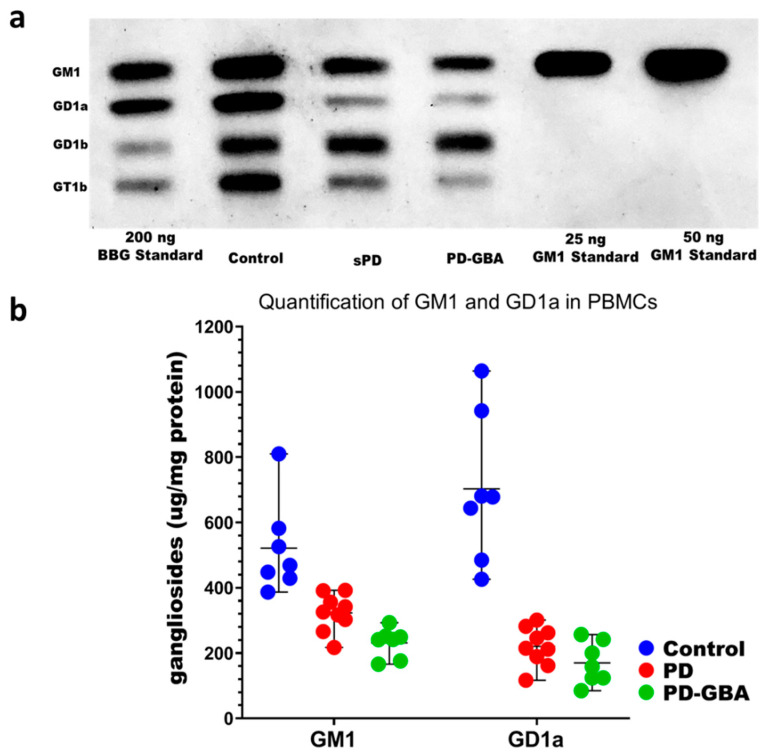
Detection and quantification of GM1 and GD1a in PBMCs. (**a**) Image of autoradiographic film depicting HPTLC separation and CtxB-HRP detection of GM1, GD1a and other ganglio-series following N’ase treatment. (**b**) GM1 and GD1a are shown as the mean and range of levels by upper and lower lines for PBMCs from seven healthy controls, nine sPD, and seven PD-GBA patients. One-way ANOVA revealed a significant difference in GM1 between controls and the other two groups: F(2, 20) = 19.45, *p* < 0.0001. Tukey’s HSD post-hoc testing revealed that sPD and PD-GBA patients both had significantly less GM1 than healthy controls (*p* = 0.0008 and *p* < 0.0001 resp.). One-way ANOVA revealed a significant difference in GD1a between controls and the other two groups: F(2, 20) = 33.81, *p* < 0.0001. Tukey’s HSD post-hoc testing revealed that sPD and PD-GBA patients both had significantly less GD1a than healthy controls (*p* < 0.0001 for both). Additionally, PD-GBA patients had lower amount of GM1 (*p* = 0.13) and GD1a (*p* = 0.74) compared to sPD patients, though these were not significant.

In our first procedure GM1 ganglioside from PBMCs was separated via high performance thin-layer chromatography (HPTLC) and detected with cholera toxin B (CtxB) subunit linked to horseradish peroxidase (HRP) (Figure 1).

The high sensitivity of CtxB-HRP permitted the detection of the limited levels of GM1 in these cells. GM1 and other ganglio-series gangliosides were previously reported to be absent (as major gangliosides) in human monocytes/macrophages; that study employed resorcinol spray for detection on thin-layer chromatography [11]. The latter report mentions the conflicting reports on the types of gangliosides present in such cells. Typical PBMCs preparations are known to include a number of cell types including T cells, and our previous studies demonstrated these to clearly express GM1 and GD1a [12].

Because of its high selectivity for GM1, the CtxB reagent could not detect other gangliosides initially, but three other members of the ganglio-series were revealed following desialylation by neuraminidase (N’ase) on the HPTLC plate (see Methods). Neuraminidase catalyzes the removal of all sialic acid attached to ganglio-series ganglioside except GM1, which is why it is the only ganglioside left to react with CtxB. In this process neuraminidase converts GD1a, GD1b, GT1a, and GT1b to GM1 and the GM1 produced by this procedure is detected by CtxB. We have confirmed that overnight treatment with neuraminidase to complete the process, which can be visualized on a HPTLC plate with CtxB reagent [13]. These too are shown in Figure 1. GD1a is one of the primary interests because of its function as a metabolic precursor and reserve pool for GM1. Quantification of GM1 and GD1a with the aid of ImageJ densitometric analysis (see Methods) revealed significantly less of both gangliosides in PBMCs from sporadic PD and PD-GBA patients, compared to age-matched controls. Importantly, there was minimal overlap between controls and either form of PD (Figure 1). On the other hand, while PBMCs from PD-GBA patients expressed lower GM1 on average than PBMCs from sPD patients this difference did not prove significant. From this, we conclude that the GM1 level in PBMCs constitutes a reliable biomarker for both forms of PD but can only suggestively distinguish PD-GBA from sPD—at least by this method (see below for revised procedure). We were able to achieve this distinction with the revised method described below.

### 2.3. Modified Method: Analysis of GM1 in PBMCs

**Figure 2:**
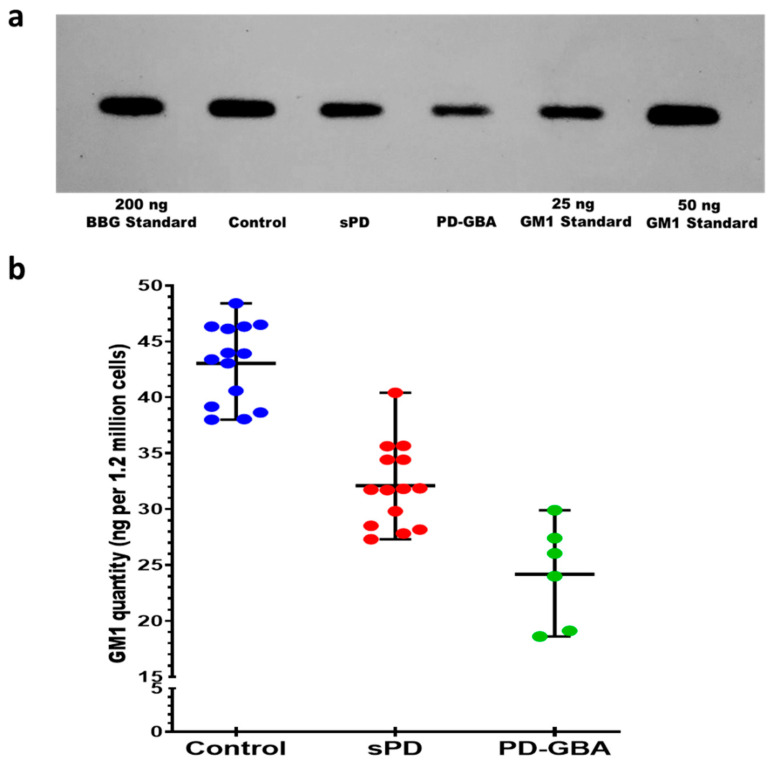
Quantification of GM1 in PBMCs. (**a**) Image of autoradiographic film depicting HPTLC separation and CtxB-HRP detection of GM1 derived from PBMCs from healthy controls, sPD and PD-GBA patients. (**b**) GM1 levels are shown as the mean represented by a solid line and range by upper and lower lines for PBMCs from healthy controls (*n* = 14), sPD (*n* = 14), and PD-GBA patients (*n* = 6). The data were normalized to the amount of lipid fraction applied for 1.2 × 10^6^ PBMCs. One-way ANOVA showed a significant difference in GM1 between groups; F(2,31) = 59.62, *p* < 0.0001. Tukey’s HSD post-hoc testing revealed that sPD and PD-GBA patients both had a significantly lower amount of GM1 compared to healthy controls (*p* < 0.0001 respectively). Additionally, PD-GBA patients had a significantly lower amount of GM1 compared to sPD (*p* = 0.0005).

Because this was a rather lengthy and involved procedure, we next attempted to modify/shorten it by omitting the N’ase application. In that case, GM1 was the only ganglioside revealed by CtxB-HRP (Figure 2). In addition to omitting N’ase, this modified procedure contained a few other relatively minor changes (see Materials and Methods).

Quantification of the available samples (six each of sPD and PD-GBA) also revealed significantly lower GM1 in both sPD and PD-GBA, and fortuitously, a significantly lower level in PD-GBA than sPD (Figure 2). Accordingly, we conclude that this modified/shortened method provides a differentiated diagnosis of sPD and PD-GBA.

### 2.4. ROC Analysis of GM1 and GD1a in the PMBCs in sPD and PD-GBA Patients and Healthy Controls

#### 2.4.1. Original Analytical Method

**Figure 3:**
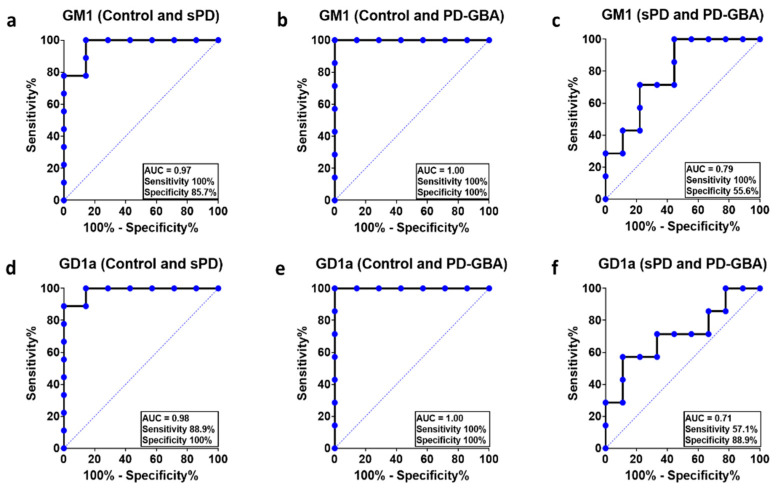
ROC curves of GM1 and GD1a in PBMCs measured using the original analytical method. The ROC curves were used to determine the probability of using GM1 expression in PBMCs to differentiate sPD (**a**) and PD-GBA (**b**) patients from controls; and PD-GBA patients from sPD patients (**c**). The same was analyzed using GD1a expression in PBMCs comparing healthy controls and sPD patients (**d**); healthy controls and PD-GBA patients; (**e**) sPD and PD-GBA patients (**f**). The area under the curve (AUC) and the sensitivity (%) and specificity (%) for cutoff levels are indicated on the plots.

Receiver operating characteristics (ROC) curves were performed to analyze the diagnostic accuracy of using ganglioside levels detectable in PBMCs of healthy controls, sPD and PD-GBA patients. The area under the curve (AUC) was statistically significant (*p* < 0.05) for GM1 when comparing healthy controls with sPD (AUC = 0.97) and PD-GBA (AUC = 1.0) patients; and GD1a when compared with healthy controls (sPD: AUC = 0.98; PD-GBA: AUC = 1.0) (Figure 3). The determination of the sensitivity and specificity of GM1 as a biomarker revealed both high sensitivity (100%) and specificity (>85.7%) in distinguishing sPD and PD-GBA patients from controls with an optimal cutoff value (calculated using Youden’s index) of 397.5 and 324.7 µg/mg protein respectively. These cutoff values are below the lowest GM1 amount from healthy controls, indicating that this assay can distinguish between samples taken from sPD and PD-GBA patients and healthy controls with high probability. The optimal cutoff value for differentiating sPD patients from PD-GBA patients using GM1 levels was 284.5 µg/mg protein with high sensitivity (100%). The sensitivity correctly identifies the sample as originating from a patient with the disease. However, given that the specificity rate was 55.6%, this indicates that the probability of incorrectly identifying a sPD sample as a PD-GBA sample is near chance using this analytical method. 

#### 2.4.2. Modified Analytical Method

**Figure 4:**
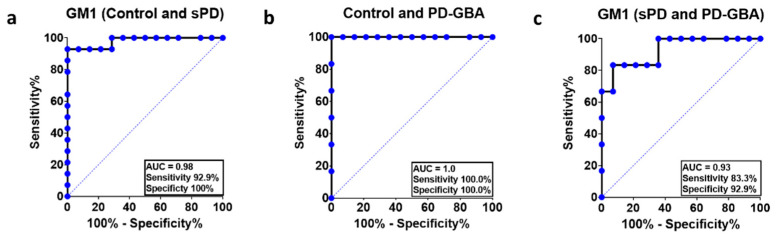
ROC curves of GM1 in PBMCs measured using the modified analytical method. The ROC curves were used to determine the probability of differentiating sPD (**a**) and PD-GBA (**b**) patients from controls; and PD-GBA patients from sPD patients (**c**) using GM1 expression levels in PBMCs. The area under the curve (AUC) and the sensitivity (%) and specificity (%) for cutoff levels are indicated on the plots.

The accuracy of the modified analytical method was evident when comparing both patient groups with healthy controls (sPD: AUC = 0.98, *p* < 0.0001; PD-GBA: AUC = 1.0; *p* = 0.0005) (Figure 4). GM1 levels in PBMCs can also be used to distinguish sPD from PD-GBA patients using the modified analytical method with better accuracy (original method: AUC = 0.79, n.s.; modified method: AUC = 0.93, *p* = 0.003). Additionally, at the optimal cutoff value of <27.62 ng, the sensitivity (>83.3%) and specificity (92.9%) of the modified analytical method can distinguish PD-GBA patients from sPD patients’ above random chance. 

The specificity of the modified analytical method (92.9%) was higher than the original analytical method (55.6%) indicating a smaller chance of incorrectly identifying a sPD patient as a PD-GBA patient, further supporting the advantage of using this modified method of GM1 measurement.

## 3. Discussion

This study revealed subnormal expression of GM1 ganglioside in PBMCs to constitute a reliable indicator of both sPD and PD-GBA. Ganglioside GD1a, revealed by N’ase application to the HPTLC plate, was also significantly deficient in sPD and PD-GBA. This was validated by assessing the diagnostic accuracy of GM1 and GD1a measurements in PBMCs using ROC curves. The high AUC values demonstrate the accuracy of these molecules for the differentiation of sPD and PD-GBA patients from controls. This was similarly robust when using our modified analytical method with simplified procedure that omitted N’ase. Additionally, the latter method proved able to distinguish between sPD and PD-GBA patients with high sensitivity and specificity. The modified analytical method had higher specificity in differentiating between sPD and PD-GBA patients than the original analytical method.

Because this study employed PBMCs from PD patients well advanced in clinical symptoms, additional work is required to substantiate this as a reliable method for diagnosis in the preclinical stage. If successful it would offer the additional advantage of also pointing to a potential disease altering treatment for PD to follow such diagnosis; this refers to GM1 replacement therapy to alleviate the neuronal deficiency of GM1 which our studies point to as the underlying cause of sPD [14].

GM1 ganglioside has been identified as a key player in the pathogenesis of PD due to its multiple regulatory and modulatory functions within the neuron [6,7]. These include—of special relevance to PD—GM1 facilitation of neuroprotection by glial cell line derived neurotrophic factor (GDNF) whose receptor complex includes GM1 as essential constituent [15]. GDNF was shown essential for the survival of adult catecholaminergic neurons, such as those of the substantia nigra [16]. It is noteworthy that NGF and BDNF, two other essential neurotrophic factors, also have receptors (TrkA, Trk B) with strongly associated GM1 [17,18]. This points to the additional neuronal types dependent on an adequate level of neuronal GM1 and is consistent with the broad array of neurons that dysfunction and gradually die, albeit at different rates, during PD progression [8]. Another GM1 function of major relevance to PD is its association with alpha-synuclein (aSyn), whose aggregation (e.g., Lewy bodies/Lewy neurites) is generally associated with and widely considered a major contributor to neuronal loss in PD. This protein was shown to bind GM1 with high affinity and specificity in a manner that maintains it in a non-aggregating conformation [19,20]. Application of GM1 to mice expressing such pathological aSyn was shown an effective means of dispersing such aggregates [21,22].

In view of these and additional vital functions of GM1 referred to above it is perhaps not surprising that even a partial deficiency of GM1 leads to gradual neuronal dysfunction and neuron death throughout the brain and body. That this would apply to virtually all neurons, albeit at different rates, would explain the enormous complexity and systemic nature of PD pathologies. It is becoming well recognized that PD does not involve selective damage to the dopaminergic neurons of the mesencephalon but is rather a severe multisystemic disorder [23]; this is well characterized, for example, in the numerous non-dopamine lesions that occur in PD [24]. Nigrostriatal neurons and those mediating the various prodromal symptoms would appear to be the most vulnerable and hence the earliest to succumb. That this is the underlying cause of sPD is supported by the mouse PD model based on mono-allelic disruption of the B4galnt1 gene which, in the process of diminishing GM2, also reduces GM1 to subnormal levels. Such mice manifest virtually all the neuropathology of PD in addition to motor dysfunctions [15,21]. The partial GM1 deficiency in such mice, caused without the use of a neurotoxin, approximates that subsequently found in human PD tissues [8]. What is particularly noteworthy is that such GM1 deficiency includes non-nervous tissues as well as neurons of the CNS and PNS, as demonstrated in the present study. PD is thus a truly systemic disorder.

In view of this model, it was speculated that human PD may have a similar genetic cause. In fact, mutated B4galnt1 has been demonstrated in patients with hereditary spastic paraplegia [25]. However, genome-wide association studies showed no evidence that this or other genes involved in GM1 synthesis are affected in sPD [26]. On the other hand, in situ hybridization histochemistry demonstrated a significant decrease in B3galt4 (GM1 synthase) and ST3gal2 (GD1a synthase) gene expression in residual neuromelanin-containing cells in the substantia nigra of PD patients; these decreases in GM1-synthesizing enzymes were cell-type specific and were not observed in non-neuromelanin containing neurons outside the substantia nigra in sPD brain [27]. Epigenetic effects, of course, are possible, such factors having been shown capable of influencing ganglioside biosynthesis [28,29]. We consider it likely a major cause of GM1 deficiency in PD derives from the aging process itself, as suggested in two key discoveries by Svennerholm’s group: (a) brain GM1 and GD1a levels vary significantly among individuals of the same age, and (b) gangliosides GM1 and GD1a both decline progressively with age [30]. This decrease was most striking for GD1a [31], the above mentioned metabolic precursor of GM1 which functions as a reserve pool for the latter through co-association with NEU3 neuraminidase [32]. We have speculated [14] that individuals whose endowed levels of GM1/GD1a fall in the very low normal range could start life with adequate levels of these two gangliosides but cross a threshold in later years when both GM1 and GD1a fall below the level necessary to maintain normal function of GDNF, aSyn and the other GM1-dependent processes. Such gradual age-related decline makes it likely that the subthreshold deficiency of GM1 characteristic of sPD would also be prevalent in the preclinical stages. An effort is underway to test this.

The promising preclinical experiments with GM1 applied to animal PD models [14] led to equally promising clinical trials with PD patients by Schneider and coworkers. This included a five-year open-label study [33] in which patients were administered an initial loading dose of 1000 mg GM1 (intravenous) followed by two subcutaneous injections of 100 mg GM1 per day. This resulted in reduced, slow linear projection of motor dysfunction based on UPDRS motor scores, and improved activities of daily living scores. It was significant that after five years the motor disability scores were lower than at baseline. This was followed by a randomized, controlled, delayed-start phase II trial that demonstrated GM1 superiority to placebo in reducing motor impairment and slowing symptom progression over a two-year period; UPDRS scores were improved at the trial end of 120 weeks compared to baseline [34]. This suggested to the investigators that “GM1 may have symptomatic and potentially disease modifying effects”. The above studies employed GM1 isolated from bovine brain and such studies were regrettably terminated due to FDA-perceived danger of contamination by prion-like proteins. Therapeutic use of *E. coli* derived GM1 proved equally effective as brain derived GM1 in a mouse PD model [35] but that too had to be discontinued due to manufacturing problems.

These examples suggest that PD origin and pathologies are causally related to the GM1 deficiencies in PD patient tissues. The present study indicates that GM1 in PBMCs serves well as a vehicle for the detection of such deficiency. PBMCs from PD patients were previously used to show a reduction of intracellular DA [36] and DA transporter [37]. What remains to be seen is whether such deficiency also occurs in preclinical PD. We have indicated our reasons for believing that such is the case, and that belief is strengthened by a recent report showing that GM1 is reduced in the serum of patients with REM sleep behavior disorder, a strong predictor of PD [38]. The latter study also demonstrated subnormal GM1 in substantia nigra, CSF, and serum. Beyond that is support by these findings that use of GM1-replacement therapy would prove truly disease modifying in a manner that would stop pathological progression. That the simplified procedure described herein also serves to distinguish between sPD and PD-GBA was an interesting additional discovery of this study.

## 4. Materials and Methods

### 4.1. Sample Collection and Patient Assessment

The PBMCs samples were provided for biochemical analysis by Roy N. Alcalay, these samples were collected during a visit to the Columbia University Irving Medical Center (NY, USA. Patients and healthy controls were assessed the same day for the presence and severity of Parkinsonian symptoms using The Unified Parkinson’s Disease Rating Scale (UPDRS). The following UPDRS exams were conducted: (1) UPDRS Part I for behavior and mood; (2) UPDRS Part II for activities of daily living; (3) UPDRS Part III motor examination.

### 4.2. PBMCs Isolation

PBMCs were isolated from the three populations mentioned above: (a) sPD, (b) PD-GBA, (c) healthy, age-matched controls—as described [39]. All PD-GBA samples were heterozygotes except one which was a homozygotic carrier. Whole blood samples were collected in PBS-diluted heparin and transferred into a Ficoll-filled Leucosep tube. This was centrifuged and the upper phase extracted and centrifuged again. The supernatant was decanted, and the cells washed. Cell counts were measured prior to resuspension and final centrifugation. The cell pellet was stored at −80 °C until shipment.

### 4.3. GM1 and GD1a Analysis

To extract total lipids, mixtures of 3.2 × 10^6^–3.8 × 10^6^ PBMC were treated with 2 mL of chloroform/methanol/H_2_O (C/M/W; 5/5/1) in a glass tube and homogenized 0.5 h with cell homogenizer and water bath sonicator. The mixture was then centrifuged at 5000 RPM for 15 min and the upper phase containing the lipids was removed from the protein-containing residue and transferred to another glass tube. The lipid solution was evaporated to dryness with a nitrogen stream and the protein quantified by a modified method of Lowry [40]; the latter results were used to determine the amount of lipid to apply to the HPTLC plate.

For HPTLC the lipid mixtures were dissolved in 55–65 µL C/M (1/2), adjusted to a volume that would allow application of 20 µL to the HPTLC plate corresponding to 100 µg protein. The Silica Gel 60 aluminum plates (Millipore Sigma, Burlington, USA; 20 × 20 cm) were placed in the paper-lined glass tank containing 150 mL C/M/0.25% aq. KCl (5/4/1) and the solvent allowed to ascend the plate for 1.5 h. The plate was dried for at least one hour and immersed in 1% polyisobutyl methacrylate (Sigma Aldrich, St. Louis, MO, USA; ave. MW 70,000—crystalline) for one minute. The plate was dried, then immersed in acetic acid buffer, pH 5.4 for one hour. The plate was immersed overnight with mild shaking in 20 mL of the same buffer containing 15 units of neuraminidase (N’ase; Clostridium—Sigma Aldrich). The plate was then washed twice for two min in phosphate-buffered saline (PBS). For blocking, the plate was immersed one hour in 15 mL PBS containing 6.7% powdered milk with mild shaking. For GM1 detection the plate was immersed one hour in 20 mL PBS containing 10 µg CtxB-HRP (Thermofisher, NY, USA), then washed 3× for 2–3 min in PBS. The plate was moved to a dark room and covered with five mL ECL Western Blotting Detection Reagent for one minute. The reagent was removed, and the plate was placed in a Hypercassette with HyBlot ES autoradiographic film and developed for five minutes. Quantification of the visible GM1 bands was carried out with ImageJ [41]. The quantities of GM1 and GD1a were determined using a ratiometric method in which the percentage change in optical density between the standards and samples was used to calculate the amount of GM1 and GD1a.

In the above procedure it was not necessary to first remove the other lipids from the gangliosides prior to HPTLC because they did not interfere due to non-reaction with CtxB; also, they generally moved well ahead of the gangliosides on the HPTLC plate.

### 4.4. Modified Analytical Method for Analysis of GM1 Alone

In this procedure we normalized the lipid quantity on the basis of cell number rather than protein content to determine the amount of lipid extract to apply to the HPTLC plate. As before, phospho- and other lipids ran well ahead of gangliosides and did not interfere. Following extraction of the lipids as described above and evaporation of the extract to dryness, C/M (1/2) was added (50–70 µL) in a calculated amount corresponding to 1.2 × 10^6^ PBMCs. In addition to omission of N’ase, other changes from the above procedure included CtxB-HRP treatment for 1.5 h and development time with HyBlot ES autoradiographic film for six minutes.

### 4.5. Statistical Analysis

The UPDRS Part I and Part II scores were combined and analyzed together. The Total UPDRS scores were also reported and is the sum of the scores from Part I, II, and III exams. For the original analytical procedure—data are shown as the average amount and range of GM1 and GD1a in PBMCs (µg ganglioside/mg protein). For the modified analytical procedure—data are shown as the average amount and range of GM1 in PBMCs (ng per 1.2 × 10^6^ PBMCs). One-way ANOVA with Tukey’s HSD post-hoc testing was used to analyze differences in ganglioside levels between controls and patient groups. The diagnostic accuracy of using ganglioside levels in PBMCs as a biomarker in sPD and PD-GBA was assessed using the receiver operating characteristic (ROC). This was completed using individual ROC curves for 3 comparisons for each biomarker (GM1 and GD1a): (1) control and sPD; (2) control and PD-GBA; and (3) sPD and PD-GBA. The area under the curve (AUC) and the optimal cutoff values for sensitivity and specificity (calculated using the Youden’s index) were further used to determine the diagnostic accuracy of these biomarkers [42,43]. All statistical analyses were completed using GraphPad Prism 9.0 (GraphPad Software, San Diego, CA, USA).

## 5. Conclusions

In this study we demonstrate that PBMCs from both sPD and PD-GBA patients express subnormal levels of GM1 and GD1a, while a modified/simplified procedure accomplishes that and also differentiates PD-GBA from sPD by demonstrating the lowest levels of GM1 in the former. Accordingly, this constitutes a promising method for early diagnosis of both forms of PD. It is clear that the benefits of early diagnosis would be significantly enhanced if accompanied by disease-altering therapy—a treatment that stops the progression of the pathology and even revives those neurons that have only become dormant/quiescent prior to final demise. We discuss the evidence suggesting that our procedure for early diagnosis points to such disease-altering therapy.

## Figures and Tables

**Table 1:** Patient demographics and clinical assessment of sPD and PD-GBA patients in Cohort 1. Data are mean ± SEM. GM1 and GD1a levels in PBMCs were measured using our original analytical method. UPDRS I and II were calculated for seven healthy controls, nine sPD, and five PD-GBA. UPDRS III and total UPDRS were calculated for seven healthy controls, nine sPD, and four PD-GBA.

	Cohort I		
Parameter	Control	sPD	PD-GBA
N	7	9	7
Sex (male/female)	2/5	6/3	2/5
Age	62.39 ± 3.58	66.28 ± 3.15	65.96 ± 2.94
Age at disease onset	N/A	57.00 ± 2.89	62.57 ± 2.81
Disease duration	N/A	9.28 ± 2.11	3.39 ± 0.78
UPDRS I and II	0.14 ± 0.14	8.56 ± 1.43	7.60 ± 3.5
UPDRS III	0.29 ± 0.18	16.89 ± 2.54	15.75 ± 3.94
UPDRS Total	0.43 ± 0.20	25.44 ± 3.11	20.25 ± 5.36

**Table 2:** Patient demographics and clinical assessment of sPD and PD-GBA patients in Cohort II. Data are mean ± SEM. GM1 in PBMCs were measured using our modified analytical method. UPDRS I and II were calculated for 12 healthy controls, 11 sPD, and four PD-GBA. UPDRS III and total UPDRS were calculated for 11 healthy controls, 11 sPD, and five PD-GBA.

	Cohort II		
Parameter	Control	sPD	PD-GBA
N	14	14	6
Sex (male/female)	6/8	9/5	3/3
Age	68.86 ± 2.25	64.38 ± 2.84	65.12 ± 2.79
Age at disease onset	N/A	57.07 ± 3.08	57.00 ± 4.28
Disease duration	N/A	7.76 ± 1.66	8.12 ± 1.93
UPDRS I and II Score	0.50 ± 0.26	7.64 ± 1.44	8.00 ± 1.29
UPDRS III Score	1.83 ± 0.72	19.45 ± 2.41	18.60 ± 5.47
UPDRS Total	2.55 ± 0.77	27.09 ± 3.31	22.00 ± 4.80

## Data Availability

The data presented in this study are available on request from the corresponding author. The data are not publicly available due to ethical reasons.

## Abbreviations

aSyn	Alpha-synuclein
AUC	Area under the ROC Curve
B3galt4	Beta-1,3-Galatosyl transferase 4; GM1 Synthase
B4galnt1	Beta-1,4-N-Acetyl-Galactosaminyltransferase 1; GM2 Synthase
BDNF	Brain-derived neurotrophic factor
CNS	Central nervous system
CSF	Cerebrospinal Fluid
CtxB	Cholera toxin B
DA	Dopamine; Dopaminergic neurons
GBA	Glucocerebrosidase
GDNF	Glial cell line derived neurotrophic factor
HPTLC	High performance thin-layer chromatography
HRP	Horseradish peroxidase
HSD	Honestly significant difference
N’ase	Neuraminidase
NEU3	Neuraminidase 3
NGF	Nerve growth factor
PBMC	Peripheral blood mononuclear cells
PBS	Phosphate-buffered saline
PD	Parkinson’s disease
PNS	Peripheral nervous system
REM	Rapid eye movement
ROC	Receiver operating characteristic
RPM	Revolutions per minute
sPD	Sporadic Parkinson’s disease
ST3gal2	Beta-galactoside alpha-2,3-sialyltransferase 2; GD1a Synthase
TrkA	Tropomyosin receptor kinase A
TrkB	Tropomyosin receptor kinase B
UPDRS	Unified Parkinson’s disease rating scale
